# Fat-containing liver lesions expanding the magnetic resonance imaging signature of hepatoblastoma

**DOI:** 10.1007/s00247-026-06698-9

**Published:** 2026-06-24

**Authors:** Gozde Ozer, Burak Dalkıran, H. Nursun Ozcan, Diclehan Orhan, Berna Oguz, Mithat Haliloglu

**Affiliations:** 1 Department of Radiology, Hacettepe University School of Medicine, Ankara, 06100 Türkiye; 2 Department of Pathology, Hacettepe University, Ankara, Turkey

**Keywords:** Children, Dual-echo imaging, Fat, Hepatoblastoma, Magnetic resonance imaging

## Abstract

**Background:**

Fat-containing liver lesions have been widely discussed in the radiology literature and include various benign and malignant lesions. However, data on the presence and imaging characteristics of intralesional fat in hepatoblastoma remain limited.

**Objective:**

To investigate the presence of intralesional fat in hepatoblastoma using dual-echo magnetic resonance imaging (MRI) and to correlate imaging findings with histopathology.

**Materials and methods:**

This retrospective study included 22 pediatric patients with hepatoblastoma who underwent pretreatment MRI with a dual-echo gradient-recalled echo sequence. Qualitative visual assessment and semiquantitative analysis using region-of-interest (ROI) measurement were performed to evaluate intralesional fat. Histopathology was accepted as the reference standard for fat detection.

**Results:**

Twenty-two patients (8 girls, 14 boys; median age, 11.5 months; range, 3-192 months) with pretreatment dual-echo MRI were included. Histopathologic evaluation demonstrated intratumoral fat in 10 of 22 tumors (45%). On MRI, intralesional fat was detected in eight lesions. Six lesions showed both histopathologic fat and signal loss on dual-echo MRI, whereas four lesions had histopathologic fat without detectable signal loss (< 1%). Two lesions demonstrated signal loss on MRI despite the absence of histopathologic fat. Using histopathology as the reference standard, MRI demonstrated a sensitivity of 60% and a specificity of 83.3% with an overall accuracy of 72.7% for detecting intratumoral fat in hepatoblastoma. Interobserver agreement for the four-level visual fat score was substantial (weighted Cohen’s *κ*=0.789).

**Conclusion:**

Intralesional fat can be present in hepatoblastoma and may be detected on MRI, expanding the recognized imaging features of this tumor.

**Graphical abstract:**

**Figure:**
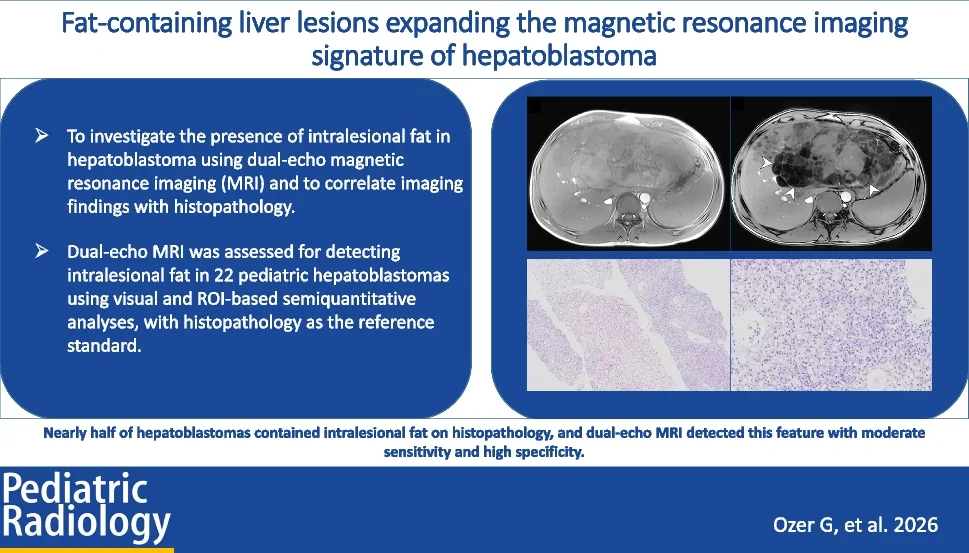

## Introduction

Primary malignant liver tumors are rare in children. Two-thirds of pediatric liver tumors are malignant, and hepatoblastoma is the most common among them [1]. Although most hepatoblastomas occur sporadically, several inherited disorders, including Beckwith-Wiedemann syndrome, familial adenomatous polyposis, and trisomy 18 are associated with increased risk [2]. Imaging plays a key role in the diagnosis and management of pediatric liver malignancies. Although ultrasound is the first choice for imaging, magnetic resonance imaging (MRI) is necessary to determine the extent of the lesion, and to characterize it when possible [3].

Fat within a liver lesion can be easily detected by dual-echo gradient-recalled echo (GRE) T1-weighted MRI sequences [4]. Chemical shift imaging exploits the difference in resonance frequency between water and fat protons to determine the relative fat content within a voxel [5]. A loss of signal on opposed-phase compared with in-phase images indicates intravoxel fat and can be used to characterize the tissue composition of liver lesions. Various benign and malignant liver lesions may contain intralesional fat, e.g., focal steatosis of the liver parenchyma, hepatocellular adenoma, and hepatocellular carcinoma (HCC) [6, 7]. Histopathologically intracellular fat is a feature of well-differentiated hepatoblastomas [8]. Although fat-containing liver lesions and the imaging features of hepatoblastoma have been well described in the literature, intralesional fat has not been widely recognized among the imaging features of hepatoblastoma.

The aim of this retrospective study was to investigate the presence and MRI characteristics of intralesional fat in hepatoblastomas using dual-echo imaging, and to correlate these findings with histopathologic evaluation.

## Materials and methods

### Patient selection

A retrospective study was performed after the approval of our institutional review board (SBA 23–361). The archives in the pediatric radiology unit were assessed for hepatoblastoma patients between 2007-2022. Only patients who underwent a pretreatment MRI that included a dual-echo GRE T1 sequence were enrolled in the study. Imaging findings, histopathological results, age at diagnosis, and serum alpha-fetoprotein (AFP) levels of the patients were recorded.

### Imaging technique

MRI scans were performed using one of two scanners: 1.5-T Signa HDxt (GE Healthcare, Milwaukee, WI) and 3-T Signa Architect (GE Healthcare, Milwaukee, WI). Although there have been some changes in scanning parameters over the years, the abdominal MR imaging protocol in our institution included breath-hold coronal fast imaging employing steady-state acquisition (FIESTA), axial single-shot fast spin echo (SSFSE), axial in- and opposed-phase chemical shift imaging, respiratory-triggered T2-weighted fast spin-echo with fat saturation, and 3D liver acquisition with volume acceleration (LAVA) sequences before and after injection of the contrast agent. Imaging parameters for chemical shift imaging at 1.5 T were as follows: TR, 160 ms; TE, 4.9 ms (in-phase) and 2.4 ms (opposed-phase); flip angle, 70°; matrix, 256×192; and slice thickness, 5 mm. For 3 T, the parameters were as follows: TR, 150 ms; TE, 2.4 ms (in-phase) and 1.2 ms (opposed-phase); flip angle, 45°; matrix, 350×300; and slice thickness, 6 mm; time of acquisition 15–17 s. Diffusion-weighted imaging (DWI) was acquired using *b*-values of 0 s/mm^2^, 300 s/mm^2^, and 600 s/mm^2^ on the 1.5-T scanner and 50 s/mm^2^ and 800 s/mm^2^ on the 3-T scanner. Extracellular gadolinium-based contrast medium (Dotarem; Guerbet, Paris, France) at a dose of 0.2 mL/kg was injected intravenously.

### Radiologic evaluation

Pretreatment MRI examinations of 22 patients were independently reviewed on a picture archiving and communication system (PACS; GE Medical Systems, Milwaukee, WI, USA) by one pediatric radiology fellow (G.O.) with 10 years of experience and one radiology resident (B.D.) with 3 years of experience in body imaging, both blinded to histopathologic results. Any disagreements were resolved by a senior pediatric radiologist (M.H.) with 30 years of experience in pediatric radiology, who made the final decision. Images were assessed for the PRETEXT stage and the presence of intratumoral fat on dual-echo imaging. Tumor size was determined by measuring the maximum diameter of the lesion in the transverse plane.

For the qualitative assessment of intratumoral fat, radiologists visually evaluated signal loss within the lesions on opposed-phase images compared with in-phase dual-echo images. A four-level visual fat score was assigned according to the estimated proportion of tumor volume containing fat:<25%, 25–49%, 50–74%, and 75–100% of the overall tumor volume.

For the semiquantitative assessment of intratumoral fat, on the axial slice showing the largest cross-sectional area of the lesion, a freehand ROI was drawn around the tumor on both in-phase and opposed-phase images. The mean signal intensity (SI_mean_) was measured on both in-phase and opposed-phase images, and the percentage signal loss between the two was calculated.

### Histopathological evaluation

The archives of the pediatric pathology department were retrospectively reviewed for histopathologic evaluation. Hepatoblastoma diagnosis and subtyping were performed according to the International Pediatric Liver Tumors Consensus Classification, which classifies hepatoblastoma as epithelial or mixed epithelial-mesenchymal, and further subdivide epithelial tumors into fetal, embryonal, small-cell undifferentiated, and mixed epithelial subtypes, and no tumors were identified as hepatocellular neoplasm-not otherwise specified (HCN-NOS) [9, 10]. All available histologic slides were assessed for fat within the tumor. The reevaluation was performed by a pediatric pathologist (D.O.) with 22 years of experience in pediatric pathology, who was blinded to all imaging findings. Histopathology was accepted as the gold standard for determining the presence or absence of intratumoral fat.

### Statistical analysis

Statistical analyses were performed using SPSS version 28.0 (IBM, Armonk, NY). The distributions of continuous variables were assessed using histograms and probability plots and the Shapiro-Wilk test. Normally distributed continuous variables were given as mean±standard deviation, and non-normally distributed variables as median (minimum-maximum). Comparisons between groups were performed using the Mann-Whitney *U* test for continuous variables and the Fisher exact test for categorical variables.

Diagnostic performance of dual-echo MRI for detecting intratumoral fat was evaluated using sensitivity, specificity, positive predictive value, negative predictive value, and accuracy, using histopathology as the reference standard. 95% confidence intervals (Clopper-Pearson) were calculated. Additionally, ROC curve analysis was performed using whole-lesion SI_mean_-derived signal loss percentage as a continuous predictor, with histopathology as the reference standard. Interobserver agreement for the four-level visual fat score was assessed between the two radiologists using weighted Cohen’s kappa.

## Results

**Fig. 1:**
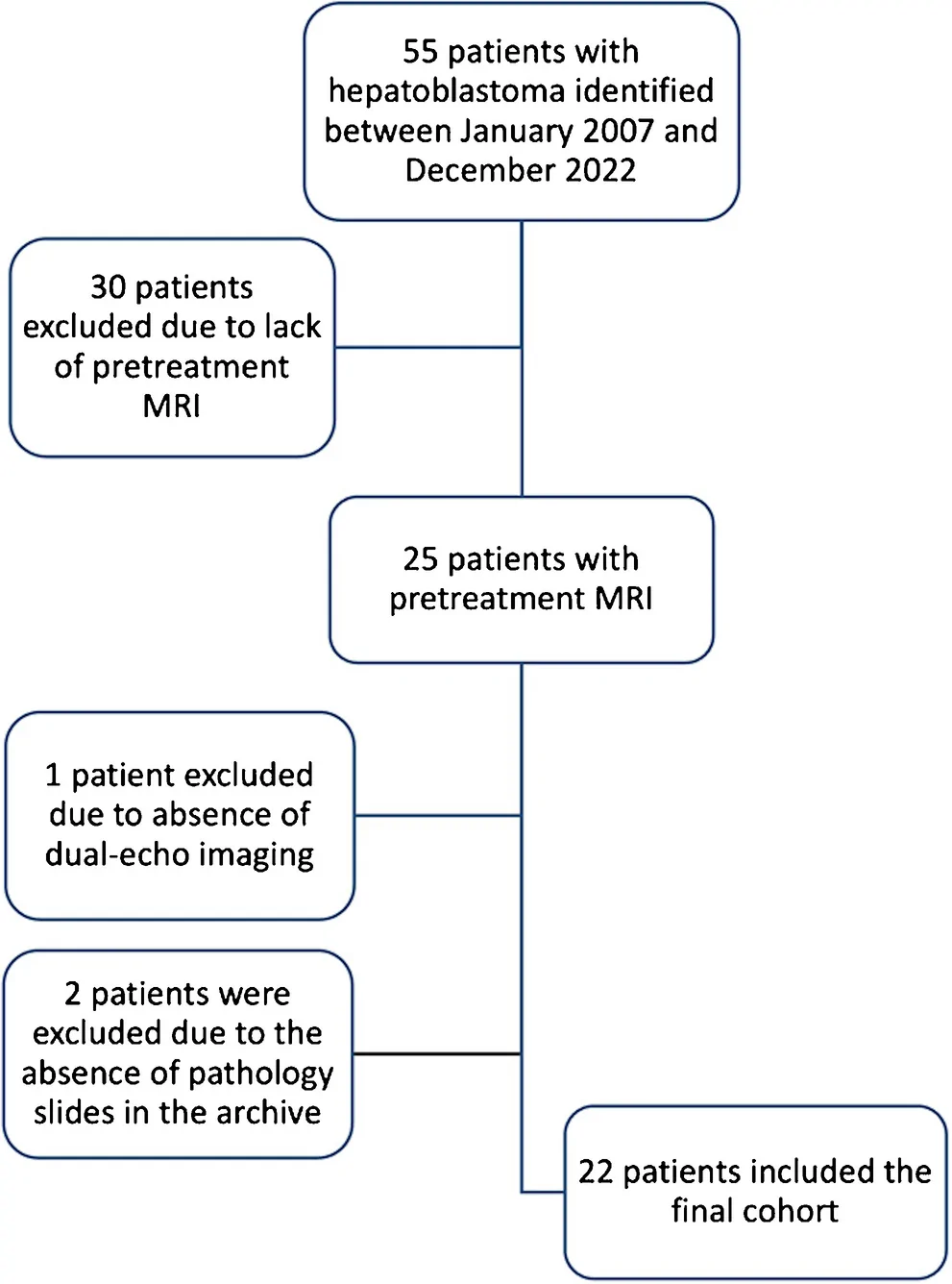
Flowchart of patient selection

Fifty-five hepatoblastoma patients with pretreatment imaging were identified. In total, 22 patients (8 girls, 14 boys; median age 11.5 months, range 3–192) with dual-echo imaging were included in the study. The patient selection flow is summarized in Fig. 1.

The median of the maximum transverse diameters of the lesions was 90 mm (range 51–190). AFP level was available for 20 patients at the initial diagnosis, and the median AFP level was 29,043 ng/dL (range 69–765,365). PRETEXT stages were distributed as PRETEXT I in one patient (4%), PRETEXT II in eight patients (36%), PRETEXT III in eight patients (36%), and PRETEXT IV in five patients (22%). Demographic features and tumor characteristics of the patients are given in Table 1.

**Table 1:** Demographic features and tumor characteristics of the patients

Sex	14 boys, 8 girls
Age at diagnosis (months)	11.5 (range 3–192)
Tumor size (maximum transverse diameter)	90 mm (range 51–190)
AFP level at diagnosis	29,043 ng/dL (range 69–765,365)
PRETEXT stage	I, 1 (4%); II, 8 (36%); III, 8 (36%); IV, 5 (22%)
Histopathologic subtype	Embryonal (*n*=7) Pure fetal (*n*=2) Mixed epithelial (*n*=10) Small cell undifferentiated (*n*=1) Mixed epithelial-mesenchymal (*n*=2)

*AFP* alpha-fetoprotein, *PRETEXT* pre-treatment extent of disease

**Fig. 2:**
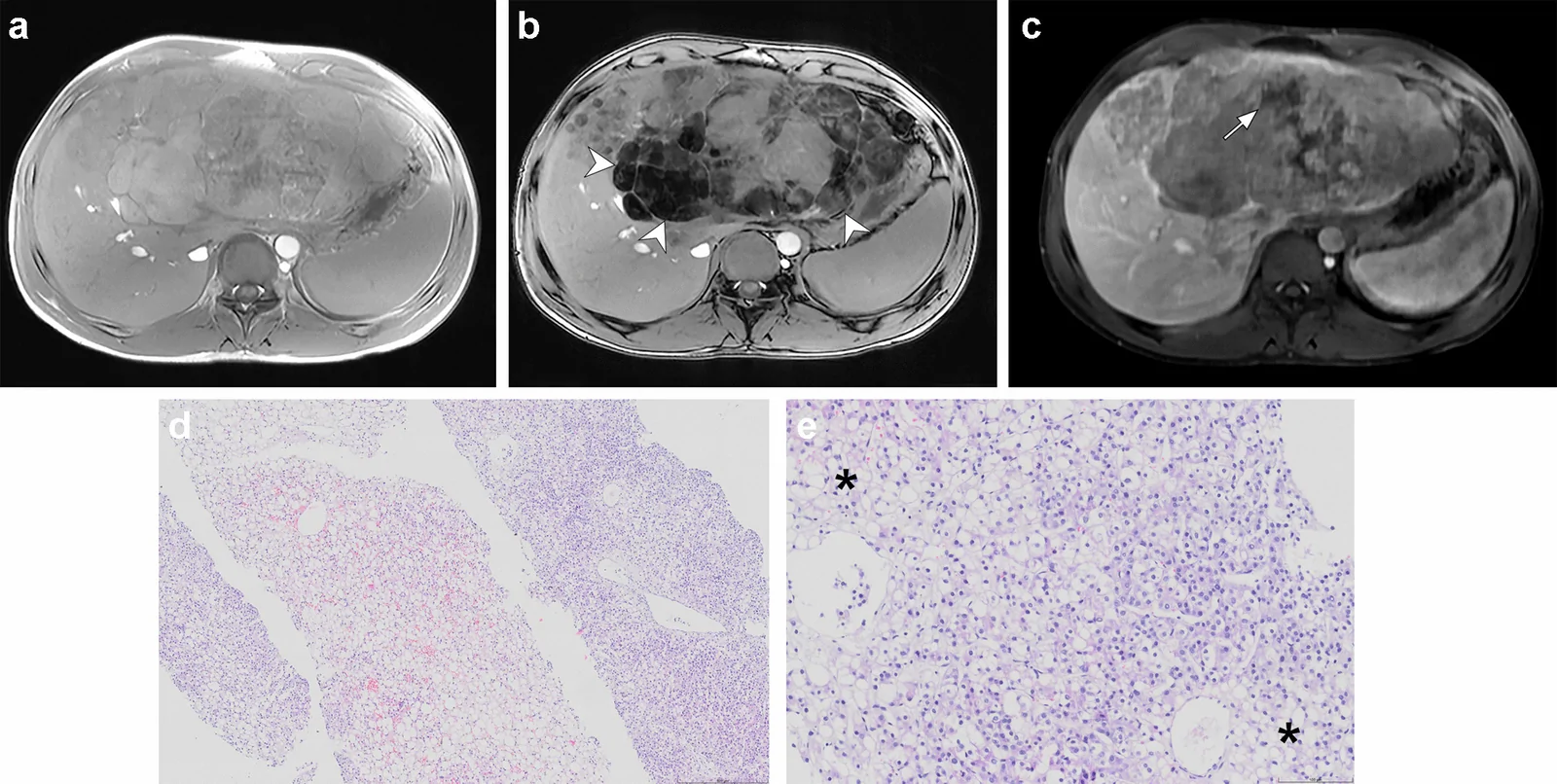
A 16-year-old boy with hepatoblastoma. **a** Axial in-phase and (**b**) opposed-phase images demonstrate intralesional fat (*arrowheads*) as decreased signal intensity on the opposed-phase image. **c** Axial post-contrast fat-saturated T1-weighted image shows a heterogeneous large mass with central necrosis (*arrow*). **d** Core biopsy of the liver mass revealing embryonal type hepatoblastoma with steatosis (hematoxylin and eosin, *scale bar* 500 µm). **e** Light microscopic examination showing hepatoblastoma with macrovesicular fat (*asterisks*) (hematoxylin and eosin, *scale bar* 100 µm)

Pretreatment pathology specimens were available for 22 patients. Histopathologically, seven tumors were classified as embryonal subtype, four tumors as pure fetal subtype, eight tumors as mixed epithelial subtype, and only one tumor as small cell undifferentiated subtype (Fig. 2). In two tumors, mesenchymal components were present in addition to epithelial components consistent with mixed epithelial-mesenchymal hepatoblastoma. Histopathologically, intratumoral fat was present in 10 of 22 tumors (45%), most commonly in the embryonal subtype (*n*=5), followed by mixed epithelial (*n*=3) and pure fetal (*n*=2). No significant differences were found between histopathologic fat-positive (*n*=10) and fat-negative (*n*=12) groups regarding sex (*P*=0.67), median age (*P*=0.12), serum AFP levels (*P*=0.79), or maximum transverse tumor diameter (*P*=0.76).

**Fig. 3:**
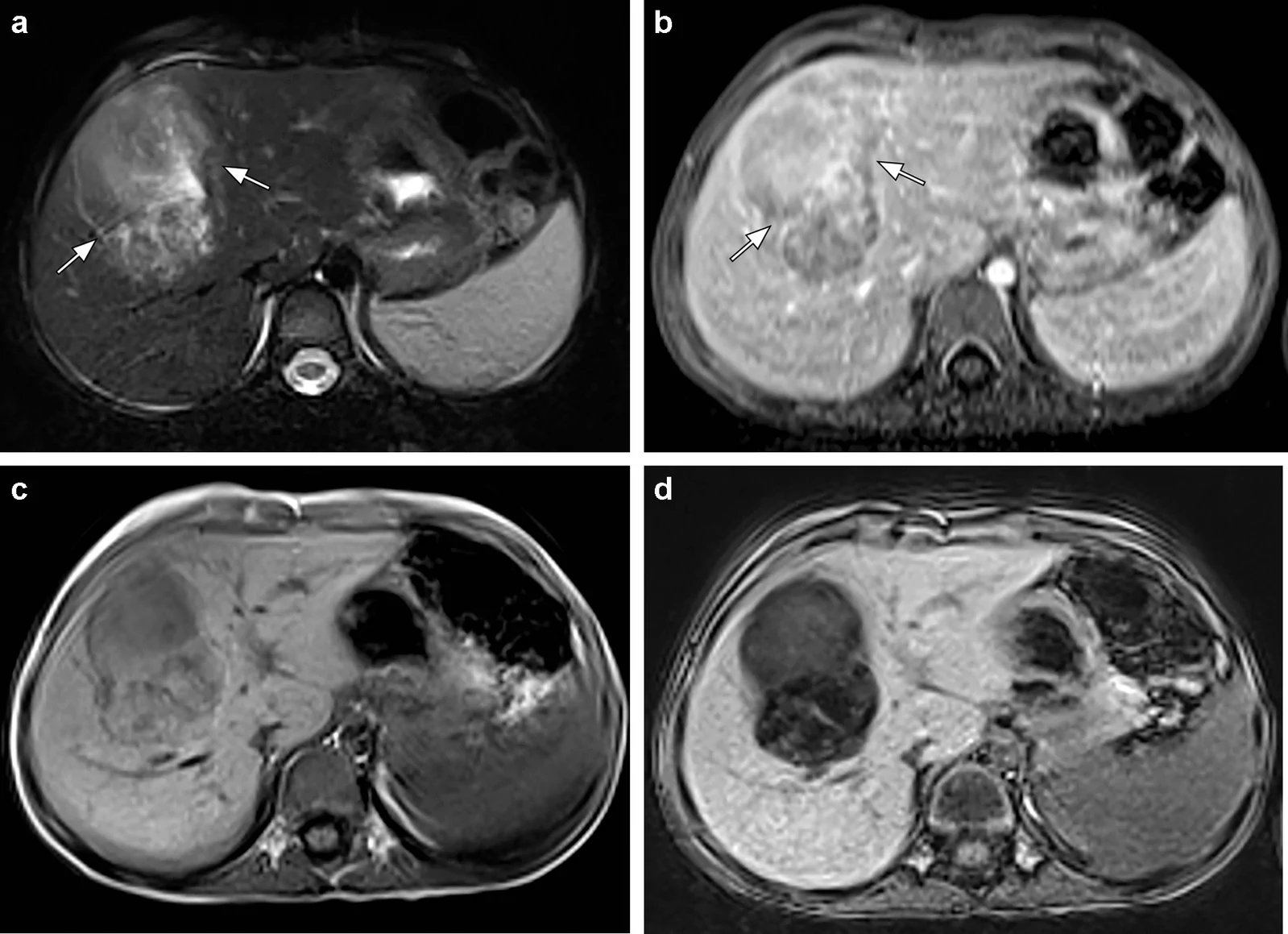
A 3-year-old boy with hepatoblastoma. **a** Axial fat-saturated T2-weighted and **b** post-contrast T1-weighted images demonstrate a large, heterogeneously enhancing mass (*arrows*) within the liver. **c** Axial in-phase and **d** opposed-phase images reveal a diffuse signal loss on the opposed-phase image throughout the entire lesion, consistent with intralesional fat

Radiologic-pathologic correlation of the lesions and SI_mean_ values are presented in Table 2. Six lesions demonstrated both histopathologic fat and signal loss on dual-echo imaging (mean signal loss percentage, 34%) (Fig. 3). Four lesions had histopathologic fat without signal loss on dual-echo imaging (mean signal loss percentage,<1%). Two patients showed signal loss on dual-echo images despite the absence of histopathologic fat (mean signal loss percentage, 14%). Ten patients demonstrated neither histopathologic nor radiologic fat (mean signal loss percentage,<1%). Using a visual scoring system, four lesions demonstrated <25% fatty area, while two lesions showed 25–49% fatty area and two lesions showed >75% fatty area. On fat-saturated T2-weighted images, two lesions showed fat suppression; these lesions corresponded to those with >75% fat on visual scoring. Interobserver agreement for the four-level visual fat score was substantial (weighted Cohen’s *κ*=0.789), with an observed exact agreement of 75% (6/8).

**Table 2:** Correlation between histopathologic fat and signal loss on dual-echo MRI

Histopathologic fat	Radiologic fat	n	Mean SI_mean_in-phase	Mean SI_mean_opposed-phase	Signal loss percentage on dual-echo MRI
+	+	6	348	229	34%
+	-	4	223	221	<1%
-	+	2	294	252	14%
-	-	10	388	386	<1%

*MRI* magnetic resonance imaging, *SI* signal intensity

Using histopathology as the reference standard, MRI demonstrated a sensitivity of 60% (95% CI 26.2–87.8) and a specificity of 83.3% (95% CI 51.6–97.9) for detecting intratumoral fat. The positive predictive value was 75% (95% CI 34.9–96.8), and the negative predictive value was 71.4% (95% CI 41.9–91.6), with an overall accuracy of 72.7% (95% CI 49.8–89.3). Receiver operating characteristic (ROC) analysis of whole-lesion SI_mean_-derived signal loss yielded an area under the curve (AUC) of 0.612 (95% CI 0.361–0.864). An exploratory optimal threshold of 4% signal loss was identified, corresponding to a sensitivity of 70% and a specificity of 66.7%.

## Discussion

Our study demonstrated that almost half of hepatoblastoma lesions may contain intralesional fat on histopathology, and MRI can detect fat with moderate sensitivity and high specificity. Hepatoblastomas are embryonal tumors arising from hepatocyte precursors. Most hepatoblastomas have a histologically epithelial origin, including fetal, embryonal, and small-cell undifferentiated types [9]. Similarly, the majority of lesions in our cohort (91%) were of the epithelial subtype. Well-differentiated fetal type hepatoblastomas are composed of tumor cells that resemble fetal hepatocytes and may contain glycogen or lipid in their cytoplasm [10]. In addition to epithelial components, one-third of hepatoblastomas may contain mesenchymal derivatives including osteoid, skeletal muscle, and cartilage, and are called “mixed epithelial-mesenchymal” hepatoblastomas [10]. Hepatoblastomas may contain either intracellular fat as cytoplasmic inclusion or macroscopic fat as a mesenchymal component [11, 12]. In our cohort, histopathologic evaluation showed that all fat-containing lesions were epithelial type, including fetal, embryonal, and mixed (fetal+embryonal) epithelial subtypes. However, the low prevalence of mesenchymal and small-cell undifferentiated subtypes in our cohort limits the ability to determine a relationship between intralesional fat and specific histopathologic subtype.

Dual-echo MRI is a widely used and reliable technique for the detection of fat in the liver and focal hepatic lesions. In our study, using histopathology as the reference standard, dual-echo MRI detected intratumoral fat with moderate sensitivity and high specificity. However, histopathologic fat was not detected on MRI in four lesions. This discrepancy may be attributed to several technical and methodological factors. First, dual-echo chemical shift imaging exhibits limited sensitivity for small-volume intravoxel lipid deposits, in which the fat fraction may be insufficient to produce detectable signal loss [13]. Second, prior studies have shown that dual-echo MRI may underestimate intralesional fat content when compared with conventional, visually assessed histopathologic evaluation [14]. Conversely, in two lesions, MRI demonstrated intralesional fat without histopathologic confirmation. In these cases, the visual MRI fat score was 25–49%, and both lesions demonstrated a mean signal intensity drop on dual-echo exceeding 4%. This discrepancy may be related to sampling limitations of tru-cut biopsy, as focal fatty components within heterogeneous tumors may not be adequately represented in limited tissue samples. In addition, technical factors such as partial volume averaging and susceptibility effects and intratumoral hemorrhage and necrosis may contribute to this discrepancy.

Intralesional fat has been reported as an ancillary MRI feature of pediatric malignant hepatocellular tumors [15]. HCC is the second most common primary malignant liver tumor in children and represents an important differential diagnosis of hepatoblastoma [16]. Although patient age at presentation and serum AFP levels can be helpful in distinguishing these entities, their imaging features may substantially overlap, particularly in older children [17]. Intralesional fat is a well-recognized imaging feature of adult HCC. Approximately 7.5% of adult HCCs demonstrate intratumoral fat on MRI, most commonly in well-differentiated tumors, and the presence of fat in a cirrhotic liver is considered highly specific for HCC [18, 19]. However, pediatric HCC may differ from adult HCC in both imaging and histopathologic characteristics. Although data on pediatric HCC are limited, Arias et al. reported intralesional fat in 22% (2/9) of pediatric HCC cases [20]. Our study demonstrates that, in addition to HCC, hepatoblastoma should be kept in mind in the differential diagnosis of fat-containing primary malignant liver tumors in children.

Although intralesional fat may be helpful in the differential diagnosis of hepatoblastoma, our results suggest that this finding should be interpreted with caution in clinical practice. Intralesional fat is not specific to hepatoblastoma and may be observed in both benign (e.g., hepatic adenoma) and malignant pediatric liver lesions (e.g., HCC), as discussed above [21]. Therefore, the presence of fat should be regarded as a neutral imaging feature rather than favoring either benign or malignant liver lesions. In addition, no significant differences were found between fat-positive and fat-negative groups in terms of demographic features, AFP levels, or PRETEXT stages in our study. Further studies with larger cohorts are needed to determine the clinical relevance of intralesional fat.

Our study has several limitations. First, the overall sample size was small because of the single-center retrospective design of the study, so results could not be extrapolated to all pediatric hepatoblastoma patients. Second, MRI data acquired from scanners with different magnetic field strengths (1.5 T and 3 T), which may have influenced lesion detection, as higher field strengths have been associated with improved spatial resolution, particularly in pediatric imaging [2]. Although both 1.5-T and 3-T MRI systems can reliably detect intralesional fat using dual-echo sequences with appropriate parameter adjustments, we acknowledge that variability in magnetic field strength across scans may have introduced minor inconsistencies in fat detectability. No subgroup or sensitivity analysis was performed to evaluate the potential impact of scanner-related differences due to the limited sample size. Third, lack of advanced fat quantification techniques, such as multi-echo proton density fat fraction (PDFF), may have limited the sensitivity for detecting intralesional fat, since signal loss on dual-echo imaging is a semiquantitative measure that does not directly correspond to a standardized fat fraction.

## Conclusion

In conclusion, intralesional fat represents an adjunct MRI finding in hepatoblastoma that may be encountered on dual-echo imaging. Recognizing this finding may provide an additional imaging clue in the differential diagnosis of pediatric liver masses.

## Data Availability

All data supporting the conclusions of this manuscript will be made available by the authors upon request.
